# Severe Malnutrition Revealing Psychotic Depression in an Elderly Patient Successfully Treated With Electroconvulsive Therapy: A Case Report

**DOI:** 10.7759/cureus.116940

**Published:** 2026-09-25

**Authors:** Um-May Sumya, Tahir Hamid

**Affiliations:** 1 General Internal Medicine, Aberdeen Royal Infirmary, Aberdeen, GBR; 2 Interventional Cardiology, Weill Cornell Medicine, Doha, QAT

**Keywords:** antidepressant, depression in elderly, electroconvulsive therapy (ect), gastroenterology and endoscopy, sertraline

## Abstract

Depression in older adults can sometimes present mainly with physical symptoms. This may delay recognition of the underlying psychiatric illness. We describe a 74‑year‑old man admitted to a medical ward with severe malnutrition caused by almost complete refusal of food. Extensive medical investigations did not reveal an organic cause. The patient was initially treated with antidepressant medication and nutritional support. However, his condition continued to deteriorate. A second psychiatric assessment identified severe psychotic depression with impaired decision‑making capacity and starvation syndrome. The patient was transferred urgently to a psychiatric unit and treated with bilateral electroconvulsive therapy (ECT). Following treatment, there was rapid improvement in mood, engagement, and oral intake. This case highlights the importance of reconsidering psychiatric diagnoses when medically unwell patients fail to improve and demonstrates the effectiveness of ECT in severe late‑life depression.

## Introduction

Depression in older adults often differs in its clinical presentation from that observed in younger populations. Rather than reporting prominent affective symptoms, older individuals frequently present with somatic complaints, including weight loss, anorexia, constipation, fatigue, and functional decline, which may initially prompt investigations for gastrointestinal, neurological, or other systemic illnesses rather than psychiatric disorders [1,2]. Consequently, severe depressive illness can be underrecognised in medical settings, resulting in delayed diagnosis and treatment.

Psychotic depression, also known as major depressive disorder with psychotic features, is a severe subtype of depression characterised by depressive symptoms accompanied by psychotic phenomena such as mood-congruent delusions and/or hallucinations, reflecting impaired reality testing. Compared with non-psychotic depression, it is associated with greater symptom severity, increased functional impairment, higher relapse rates, and poorer overall outcomes. Furthermore, psychotic depression carries a substantial mortality burden. An 18-year follow-up study demonstrated significantly higher all-cause mortality among individuals with psychotic depression (adjusted hazard ratio (aHR): 1.59) and more than a twofold increased risk of suicide (aHR: 2.36), particularly during the early period following diagnosis [3]. Patients may exhibit profound social withdrawal, nihilistic beliefs, marked reduction in oral intake, and self-neglect, which can progress to severe malnutrition, starvation syndrome, and life-threatening medical complications [4].

Electroconvulsive therapy (ECT) remains one of the most effective treatments for severe depression, particularly when psychotic features, treatment resistance, or life-threatening complications are present [5,6]. ECT induces a controlled therapeutic seizure that is thought to enhance neuroplasticity, modulate neurotransmitter systems, and restore functional connectivity within brain networks involved in mood regulation [7]. Evidence indicates that ECT produces rapid and robust clinical improvement in late-life depression and is generally safe and well tolerated in older adults when administered with appropriate monitoring and multidisciplinary support [8,9].

We present the case of a 74-year-old man admitted to a medical ward with malnutrition secondary to near-complete refusal of food intake. Despite extensive medical investigations and initial treatment with antidepressants and nutritional support, his condition continued to deteriorate. A subsequent psychiatric assessment identified severe psychotic depression with features of starvation syndrome, leading to urgent treatment with bilateral ECT and marked clinical recovery. This case highlights the importance of reassessing psychiatric diagnoses in medically unwell older adults who fail to improve as expected and demonstrates the potential life-saving role of ECT in severe late-life psychotic depression.

## Case presentation

A 74-year-old retired engineer with a history of hypertension and recurrent depressive disorder was admitted to a general medical ward with significant weight loss, poor oral intake, and refractory constipation. His depression had been well controlled in the community with sertraline 200 mg daily for approximately five years, without previous psychiatric admissions. Five months before admission, he abruptly discontinued sertraline because of worsening gastroesophageal reflux symptoms.

Following cessation of antidepressant therapy, he experienced progressive deterioration in mood and functioning. Family members described him as previously active and sociable, enjoying gardening, family activities, time with his grandchildren, and shared meals. Over subsequent months, he became increasingly withdrawn, lost interest in personal care and daily activities, and developed severe anorexia. Although he had initially lost approximately 2 kg before discontinuing sertraline, he subsequently lost a further 18 kg, while investigations for an organic cause were undertaken. Daily caloric intake was estimated to have fallen to approximately 300 kcal.

On admission, he presented with malnutrition, persistent constipation despite intensive bowel management, and symptoms of oesophagitis and acid reflux. Mental state assessment revealed profound depressive symptoms including low mood, anhedonia, hopelessness, health-related anxiety focused on bowel function, and poor engagement with treatment. He frequently refused food, fluids, and medical interventions. He expressed passive thoughts of death without active suicidal intent and demonstrated psychotic symptoms, including nihilistic beliefs and paranoid ideas that staff intended to set fire to his house. There was no history of substance misuse, significant psychological trauma, or family history of psychiatric illness.

Physical and neurological examinations were unremarkable. Initial laboratory investigations, including full blood count, renal function, thyroid function, and liver function tests, showed no major abnormalities apart from biochemical features consistent with malnutrition, including hypokalaemia (2.9 mmol/L), hypophosphataemia (0.6 mmol/L), hypoalbuminaemia (34 g/L), and low total protein levels. Computed tomography (CT) of the chest, abdomen, and pelvis demonstrated no evidence of malignancy. Upper gastrointestinal endoscopy was normal, while CT of the brain showed only a chronic lacunar infarct without acute pathology. At the beginning of admission, his poor oral intake was attributed primarily to gastrointestinal pathology, including gastro-oesophageal reflux disease and gastritis. Sertraline was restarted at 50 mg, and mirtazapine 15 mg was introduced to enhance mood and appetite; however, no meaningful improvement in oral intake or nutritional status was observed. Given progressive weight loss and malnutrition, nutritional support was escalated to total parenteral nutrition (TPN). During his medical admission, he received regular psychiatric reviews and olanzapine 2.5 mg nightly was added. Sertraline was increased to 200 mg once daily (OD) and mirtazapine 30 mg OD; psychiatric admission and electroconvulsive therapy (ECT) were not initially recommended. At admission, his weight was 53.8 kg (BMI: 17.5 kg/m²). Despite pharmacological treatment, his weight fell to 48.2 kg (BMI: 15.7 kg/m²), representing severe malnutrition. Following TPN, his weight increased modestly to 53.2 kg (BMI: 17.0 kg/m²), although significant depressive symptoms and food refusal persisted.

A second psychiatric opinion was subsequently requested because of ongoing clinical deterioration despite antidepressant treatment and nutritional supplementation. Comprehensive psychiatric reassessment concluded that the patient was experiencing severe psychotic depression. Features included profound anhedonia, psychomotor retardation, hopelessness, impaired concentration, sleep disturbance, nihilistic thinking, intermittent suicidal ideation, and psychotic beliefs regarding food, hydration, and bodily functioning. His markedly reduced nutritional intake was considered secondary to depressive illness rather than a primary eating disorder.

Mental state examination demonstrated a frail man with a nasogastric tube in situ. Speech was slowed and monotonous. Mood was objectively and subjectively depressed, with marked hopelessness and loss of pleasure. Thought processes were coherent but notable for nihilistic and paranoid beliefs. He believed that drinking water would paradoxically worsen thirst and feared that hospital staff intended to harm him. No formal thought disorder or perceptual abnormalities were identified. Decision-making capacity was significantly impaired by depressive and psychotic symptoms. Given the life-threatening consequences of his illness, including severe malnutrition requiring TPN and nasogastric feeding, he was detained under mental health legislation and transferred to an old age psychiatry unit. Because he lacked capacity to provide informed consent for ECT, multidisciplinary discussions were undertaken involving the patient, family members, psychiatric team, nursing staff, and Mental Health Officer. A Short-Term Detention Certificate was implemented, and urgent modified bilateral bitemporal ECT was commenced.

Concurrent treatment with sertraline 200 mg daily, mirtazapine 30 mg nightly, and olanzapine 2.5 mg nightly was continued. Although the first ECT session failed to induce a therapeutic seizure, nursing staff observed subtle behavioural improvements. Following subsequent effective treatments, progressive improvement became evident. The patient became increasingly engaged with staff, interacted more readily with family members, and demonstrated gradual improvement in oral intake. By the fifth treatment session, psychotic symptoms had largely resolved, appetite had returned, and nasogastric feeding was no longer required. He reported renewed enjoyment of food, increased hopefulness, and improved social interaction.

ECT was generally well tolerated. Mild short-term memory impairment was reported later in the treatment course. During the sixth treatment session, cardiac monitoring demonstrated approximately one second of asystole followed by transient sinus bradycardia of approximately 38 beats per minute during recovery. Both events resolved spontaneously without intervention, and no ongoing cardiac complications occurred (Table 1). 

**Table 1:** Electroconvulsive Therapy (ECT) Treatment Course, Seizure Parameters, Adverse Effects, and Clinical Response Across Six Treatment Sessions NG: nasogastric.

ECT session	Seizure activity	Adverse effects	Clinical effect
1	Modified bilateral bitemporal ECT. Stimuli 50, 100 and 175 mC; no observed or EEG seizure.	No immediate complication.	No therapeutic seizure. Slight early behavioural improvement only, with nursing staff noting that he got out of bed and spent time in the day room.
2	225 mC stimulus; EEG seizure observed 20 s.	No specific complication documented.	Early improvement in interaction; he asked about his grandchildren, although oral intake and NG feeding tolerance remained poor.
3	450 mC; observed seizure 51 seconds, EEG seizure 84 seconds.	No immediate complication; prolonged EEG seizure.	Brighter and more communicative; accepted medication, drank with encouragement, and tolerated NG feeding.
4	450 mC; observed seizure 24 seconds, EEG seizure 44 seconds.	No immediate complication.	Further improvement in engagement, mobility, facial reactivity, medication acceptance, and limited oral intake; residual low mood and anxiety persisted.
5	450 mC; observed seizure 49 seconds, EEG seizure 78 seconds.	No immediate complication; prolonged seizure activity.	Marked improvement in mood, appetite, oral intake, humour, insight, and social interaction; no psychotic symptoms were evident.
6	450 mC; observed seizure 26 seconds, EEG seizure 46 seconds.	Brief asystole and transient bradycardia, both self-resolving; minor memory impairment.	Full clinical remission with euthymic mood, restored appetite, improved weight, bowel function, insight, and enjoyment of activities.

Following six ECT treatments, the patient achieved clinical remission. Mood normalised, psychotic symptoms resolved, insight returned, and nutritional intake improved substantially. His weight increased from 48.2 kg (BMI: 15.7 kg/m²) at its nadir to 58.4 kg (BMI: 19.1 kg/m²) following completion of ECT. He was discharged on sertraline 200 mg daily, mirtazapine 30 mg nightly, and olanzapine 2.5 mg nightly with ongoing outpatient psychiatric follow-up.

Following completion of ECT and discharge from hospital, patient demonstrated sustained and progressive recovery. At first outpatient review mental state examination and collateral history from his wife confirmed stable euthymia with significant improvement in depressive symptoms. His appetite had returned to normal, sleep was good, bowel function had normalised, and his weight had increased to 59 kg. He had resumed social and recreational activities, including playing cricket and planning outings with friends and family. No medication-related adverse effects were reported. At follow-up five months post-discharge, he remained in remission with no evidence of depressive or psychotic relapse. His wife reported that he had returned to his premorbid baseline, while his granddaughter affectionately described him as “Grandad 2.0,” reflecting a marked restoration of his mood, personality, and overall functioning. At a further review 10-month post-discharge, he continued to remain clinically well on sertraline 200 mg daily, mirtazapine 30 mg nightly, and olanzapine 2.5 mg nightly. Mental state examination remained normal, with no depressive or psychotic symptoms. His weight had increased further to 12.8 stone (81.3 kg). Cognitive assessment using the Montreal Cognitive Assessment (MoCA version 8.1) yielded a score of 26/30, with points lost only on delayed recall, indicating no significant cognitive impairment despite mild subjective memory concerns. Overall, serial follow-up assessments demonstrated sustained remission, resolution of psychotic symptoms, restoration of nutritional status and social functioning, and a return to his usual personality and level of independence. Family members consistently reported substantial improvement, and he continued to do well on maintenance pharmacological treatment.

## Discussion

This case demonstrates how severe depression in older adults can present predominantly with physical symptoms, resulting in delayed recognition of the underlying psychiatric disorder. Older patients frequently present with anorexia, weight loss, constipation, fatigue, and functional decline rather than overt psychological complaints, often prompting extensive investigations for gastrointestinal, neurological, or systemic disease before a psychiatric diagnosis is considered [1,2]. Although exclusion of organic pathology is appropriate, prolonged focus on physical explanations may delay timely recognition and treatment of severe depressive illness.

Psychotic depression is associated with substantial morbidity, functional impairment, and increased mortality compared with non-psychotic depression [3]. Mood-congruent delusions, hopelessness, profound anhedonia, and impaired reality testing can contribute to self-neglect and refusal of food and fluids. In severe cases, these symptoms may progress to starvation syndrome, severe malnutrition, and life-threatening medical complications [4]. The present case illustrates this phenomenon, with profound depressive and psychotic symptoms initially masquerading as a predominantly gastrointestinal presentation. Despite comprehensive investigation, no organic cause adequately explained the severity of the patient's deterioration, highlighting the importance of maintaining a broad differential diagnosis in older adults with unexplained weight loss and functional decline.

Electroconvulsive therapy (ECT) remains one of the most effective treatments for severe depressive illness, particularly in patients with psychotic depression, treatment-resistant depression, catatonia, or when a rapid clinical response is required because of significant medical risk [5]. Contemporary modified ECT has demonstrated high efficacy and an acceptable safety profile, including in older adults with significant physical comorbidity [6,7]. Although transient adverse effects such as headache, nausea, confusion, and short-term memory impairment may occur, serious complications are uncommon when appropriate monitoring and multidisciplinary support are provided [6]. Furthermore, cognitive effects are usually temporary, with most patients returning to baseline cognitive functioning following recovery [8].

Several studies have reported particularly high response and remission rates for late-life depression treated with ECT, especially when psychotic features are present [6,7,9]. ECT is therefore recommended in situations where rapid improvement is required because of severe malnutrition, dehydration, refusal of nutrition, or other life-threatening consequences of depression [5,10]. In addition to improving depressive symptoms, ECT has been shown to enhance functional status, social engagement, nutritional intake, quality of life, and performance of activities of daily living, outcomes that are particularly important in frail older adults [7,9].

The clinical course of this patient was notable for limited improvement despite optimisation of antidepressant therapy, augmentation with antipsychotic medication, and intensive nutritional support, including total parenteral nutrition. The key turning point was reassessment by a second psychiatric team, which identified severe psychotic depression as the primary driver of the patient's clinical deterioration. Following initiation of ECT, the patient experienced progressive improvement in mood, oral intake, social interaction, insight, and physical functioning, ultimately achieving sustained remission. The marked weight gain, restoration of independence, resolution of psychotic symptoms, and preservation of cognitive function during follow-up are consistent with published evidence supporting the effectiveness of ECT in late-life psychotic depression [6,7,9,11,12].

This case also highlights the potential influence of diagnostic anchoring, a cognitive bias whereby clinicians continue to focus on an initial diagnostic hypothesis despite emerging evidence that may suggest an alternative explanation [13,14]. The patient's presentation was initially interpreted primarily through a gastrointestinal and nutritional lens, which was understandable given his severe constipation, reflux symptoms, and weight loss. However, failure to improve despite appropriate medical investigation and treatment ultimately necessitated reconsideration of the diagnosis. For medically complex patients who do not progress as expected, repeated multidisciplinary review and specialist reassessment are essential to avoid missed or delayed diagnoses and to ensure that potentially reversible conditions receive appropriate treatment.

## Conclusions

This case highlights the importance of maintaining a broad differential diagnosis and reconsidering the working diagnosis when an older adult with complex medical needs fails to improve despite appropriate investigation and treatment. Severe psychotic depression may present predominantly with physical symptoms, including anorexia, weight loss, functional decline, and self-neglect, mimicking gastrointestinal, neurological, or other systemic disease. In our patient, extensive medical investigations failed to identify an organic cause that adequately explained the severity of his deterioration. Subsequent psychiatric reassessment identified severe psychotic depression as the primary underlying disorder, prompting urgent treatment with electroconvulsive therapy (ECT).

The patient's remarkable recovery following ECT was characterised by resolution of psychotic and depressive symptoms, restoration of oral intake, significant weight gain, improved mobility and self-care, enhanced engagement with family and healthcare staff, and sustained recovery of social and functional independence. Follow-up over the subsequent year demonstrated continued remission, preserved cognitive function, and a return to his premorbid level of functioning.

This case reinforces the value of multidisciplinary reassessment and specialist psychiatric input in medically complex older adults with unexplained clinical deterioration. It also demonstrates that ECT can be a highly effective and potentially life-saving intervention in severe late-life psychotic depression, particularly when conventional treatments have failed or when rapid improvement is required because of life-threatening complications such as malnutrition and starvation. Early recognition and timely treatment can lead to substantial clinical, functional, and psychosocial recovery, even when the initial presentation closely resembles an organic medical illness.
